# Intercropping with Gramineous Plants in Nutrient Solutions as a Tool to Optimize the Use of Iron in *Brassica oleracea*

**DOI:** 10.3390/plants14142215

**Published:** 2025-07-17

**Authors:** Teresa Saavedra, Maribela Pestana, João Costa, Paula Gonçalves, David Fangueiro, José Paulo Da Silva, Pedro José Correia

**Affiliations:** 1 MED—Mediterranean Institute for Agriculture, Environment and Development & CHANGE—Global Change and Sustainability Institute, Faculty of Science and Technology, Building 8, Universidade do Algarve, *Campus* of Gambelas, 8005-139 Faro, Portugal; fpestana@ualg.pt (M.P.); pcorreia@ualg.pt (P.J.C.); 2 Centre of Marine Sciences (CCMAR/CIMAR LA), Universidade do Algarve, *Campus* of Gambelas, 8005-139 Faro, Portugal; jpsilva@ualg.pt; 3 Faculty of Science and Technology, Building 8, Universidade do Algarve, *Campus* of Gambelas, 8005-139 Faro, Portugal; costajoaomiguel@gmail.com; 4 LEAF—Linking Landscape, Environment, Agriculture and Food Research Center, Instituto Superior de Agronomia, Universidade de Lisboa, Tapada da Ajuda, 1349-017 Lisboa, Portugal; pgoncalves@isa.ulisboa.pt (P.G.); dfangueiro@isa.ulisboa.pt (D.F.)

**Keywords:** iron deficiency, nutrient uptake, perennial grasses, photosynthetic efficiency, SPAD-values

## Abstract

This study aimed to evaluate the impact of intercropping *Brassica oleracea*. with three perennial grasses (*Poa pratensis* L., *Lolium perenne* L., and *Festuca rubra* L.) under varying levels of iron (Fe) availability (Fe0, Fe1 and Fe5) in nutrient solutions. The research focused on biomass accumulation, photosynthetic efficiency, root development, nutrient uptake, and oxidative stress response. In the absence of Fe, *Brassica* sp. exhibited chlorosis, reduced biomass, and increased ferric chelate reductase (FCR) enzyme activity as an adaptive response. *Brassica* plants intercropped with *Poa* sp. maintained higher chlorophyll (Chl) levels and photosystem II efficiency (F_v_/F_m_ values), mitigating Fe deficiency effects. Catalase activity and polyphenol production varied with intercropping species, indicating differential stress response mechanisms. Intercropping improved Zn, Mn, and P accumulation, with *Poa* sp. facilitating greater Zn and Mn uptake. Intercropping *Brassica* sp. with specific grass species offers potential agronomic benefits by improving Fe use efficiency, mitigating stress, and enhancing nutrient uptake. Future research should focus on optimizing intercropping combinations for sustainable agricultural practices.

## 1. Introduction

Iron (Fe) is an essential micronutrient for plants and is a fundamental component of numerous biological processes, including redox reactions, chlorophyll (Chl) synthesis, photosynthesis, and respiration [1].

Iron deficiency leads to a decrease in the concentration of photosynthetic pigments in leaves, a condition known as iron chlorosis. This condition primarily affects young leaves, manifesting as interveinal chlorosis accompanied by fine reticulation [2]. Iron chlorosis is a major limiting factor for fruit trees established on calcareous soils in Mediterranean areas due to limitations in iron absorption and/or utilization by plants, and/or long-distance transport. It affects several metabolic processes and leads to nutrient imbalances in plants [3]. It is also responsible for significant decreases in yield, fruit size, and quality, as well as delaying fruit ripening [4].

When faced with Fe depletion, plants activate a set of mechanisms that can be grouped into two distinct strategies for iron acquisition [5]: Strategy I, also known as the reducing strategy, and Strategy II, otherwise referred to as the complexant strategy. Strategy I, typical of dicotyledonous plants and non-gramineous monocot plants [6], involves rhizosphere acidification, ferric reduction via membrane-bound ferric reductases (FROs), and subsequent uptake of Fe^2+^ through specific transporters such as IRT1 [7,8]. In contrast, gramineous species employ Strategy II, which relies on the biosynthesis and secretion of phytosiderophores (PS) that chelate Fe^3+^, enhancing its uptake via specific transporters like TOM1 [9,10]. Rice plants have been found to produce and secrete PS, also possess a root Fe(II) uptake system called OsIRT1, and exhibit Fe(III) reduction like Strategy I plants [11,12,13]. Furthermore, other grasses have been shown to produce PS or related compounds and to manifest elevated ferric chelate reductase (FCR) activity under Fe deficiency in aerobic soils [10,14,15].

The utilization of grass species in orchards is a common practice, with the primary function being to enhance soil fertility [16,17,18,19]. According to [20], significant chemical diversity in root exudates was exhibited by various grass species in response to environmental factors in field plots. For instance, the species *Festuca rubra* L., *Lolium perenne* L. and *Poa pratensis* L. are perennial grasses that are typically intercropped with fruit trees on calcareous soils [17]. Several studies have described how intercropping systems can improve the Fe nutrition of crops, which usually involves intercropping Strategy I plants with Strategy II (gramineous) species [19,21,22]. For example, intercropping is useful in mitigating Fe deficiency in citrus, grapevine and olive crops, as plants use mechanisms to improve Fe availability in the rhizosphere by releasing PS, organic acids and other compounds with the ability to complex Fe [19,23]. As stated by Dai et al. [24], the integration of peanut and maize intercropping crops has the potential to enhance the Fe nutrition of peanuts in calcareous soils.

The recovery of Fe-chlorotic plants was observed following the foliar applications of a plant extract containing *Poa pratensis* L., *Lolium perenne* L., and *Festuca rubra* L. species from grass clippings [25,26]. Furthermore, a recent study by our group [27] demonstrated that these grass species exhibit Fe-deficiency responses, such as a reduced root Fe levels and increased levels of small peptides and/or peptide derivatives with Fe chelating properties, which were previously attributed mainly to dicots. These findings suggest a broader diversity of Fe acquisition responses within Strategy II plants.

Additionally, extracts obtained from grasses have been shown to mitigate and aid recovery from Fe chlorosis in strawberry plants [25,26], encouraging further investigation into compounds involved and their effects on intercropped horticultural species.

The principal objective of the present study was to determine whether *Brassica* sp. plants cultivated under different Fe concentrations in nutrient solutions benefit from intercropping with gramineous plants that employ a distinct Fe acquisition strategy. The specific objectives of this study were: (i) to assess the morphological and physiological responses of *Brassica* sp. plants under different Fe levels in nutrient solutions, and to evaluate the potential advantages of intercropping with grass compared to a monoculture system.

## 2. Results

### 2.1. Chlorophyll Concentration

**Figure 1:**
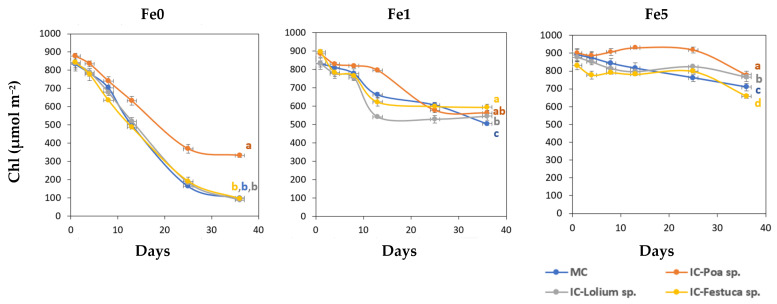
Total leaf chlorophyll concentration (Chl, µmol m^−2^) of young leaves of *Brassica oleracea* for the different treatments during the experimental period (Mean and standard error). Fe0: 0 µM of Fe in nutrient solution; Fe1: 1 µM of Fe in nutrient solution and Fe5: 5 µM of Fe in nutrient solution. MC-Monocrop and IC-Intercrop. At the final date (36 days), means with the same letter were not significantly different (*p* < 0.05; Duncan multiple range test).

Figure 1 shows the variation in the Chl values over time in the young leaves obtained from the treatments Fe0, Fe1 and Fe5.

On day 8, the first symptoms of Fe chlorosis appeared in the plants of the Fe0 treatments. The leaves turned light yellow and SPAD values decreased around 25%. After day 13, the *Brassica* sp. plants grown together with *Poa* sp. without Fe (IC-*Poa* sp.), showed higher total Chl values than the other intercropping treatments. On the other hand, plants of Fe1 treatments displayed the first symptoms of Fe deficiency on day 13. By the final date, *Brassica* sp. plants in the MC system showed significantly lower Chl values compared to those in the IC system. Under non-limiting Fe conditions (Fe5), Chl values of *Brassica* sp. plants intercropped with *Poa* sp. were significantly higher than the other treatments on the last day, while the lowest value was recorded in the *Brassica* sp. and *Festuca* sp. system.

As illustrated in Figures S1 and S2, the general aspect of *Brassica* plants cultivated in monoculture (MC) and intercropping (IC) with three grass species (*Poa* sp., *Lolium* sp. and *Festuca* sp.) can be observed, along with three Fe levels in nutrient solutions (0, 1 and 5 µM Fe, respectively: Fe0, Fe1 and Fe5).

Severe chlorosis, characterized by yellowing of the upper leaves, was observed in plants cultivated in the absence of Fe in the nutrient solution. However, this chlorosis was mitigated in *Brassica* plants grown in the presence of *Poa* (Poa-Fe0). On the other hand, plants grown in Fe5 had all green leaves without chlorosis, regardless of whether they were monocultures or grown with intercropped grasses. The Fe1 treatments exhibited slight chlorosis and interveinal yellowing in the upper leaves of the *Brassica* plants.

### 2.2. Growth Parameters

*Brassica* plants cultivated within the intercropping system (IC) in the presence of Fe (Fe1 and Fe5 treatments) exhibited greater heights in comparison to those cultivated in the absence of Fe (monoculture or intercropped) (Table 1). Specifically, the plants in Fe1 and Fe5 treatments were taller when intercropped with *Lolium* sp. or *Festuca* sp., with values ranging from 18.7 to 19.7 cm, compared to values > 16 cm in the remaining treatments. The plants from the Fe5 treatment in both systems (MC and IC) showed a greater number of leaves (means between 10 and 11) compared to plants with the lowest levels of Fe. Additionally, the *Brassica* sp. plants in the Fe1 treatment also exhibited a greater number of leaves, but only when intercropped with *Festuca* sp. (10.7 ± 0.2).

*Brassica* sp. plants intercropped with *Lolium* sp. or *Festuca* sp. and in the presence of Fe, showed higher values of root DW. The DW of the shoot part was higher in the Fe5 treatment in consociation with the *Lolium* sp. Regarding the root/shoot ratio, *Brassica* sp. plants that grew in consociation with the *Lolium* sp.in the absence of Fe (Fe0) and in consociation with *Festuca* sp. with 1 µM in the nutrient solution, exhibited a significantly higher average value (0.4 ± 0.02). In contrast, the ratio values observed in the other treatments ranged from 0.2 to 0.3.

### 2.3. Physiological Parameters

The *Brassica* sp. plants grown in intercropping with the *Poa* sp. in the Fe5 treatment had higher total Chl values in the young leaves (Table 2).

In the MC system, *Brassica* sp. plants grown in Fe1 or Fe5 conditions exhibited a F_v_/F_m_ ratio of 0.82 and 0.83, respectively. These values were comparable to those observed in *Brassica* sp. plants that consistently grew with Fe5, regardless of the IC species. Furthermore, the *Brassica* sp. plants from the Fe1 treatment exhibited similar values with IC with the *Poa* sp. (0.84 ± 0.01). In contrast, the F_v_/F_m_ ratio was lower in the other treatments, ranging from 0.16 (Fe0-*Lolium* sp.) to 0.69 (Fe1-*Festuca* sp.).

The basal fluorescence (F_0_) exhibited a significantly higher average value in *Brassica* sp. plants cultivated in the Fe0 treatment IC with *Poa* sp. (1791.0 ± 165.0). Conversely, the maximum fluorescence (Fm) value was found to be greater in *Brassica* sp. plants grown in consociation with the *Lolium* sp., in Fe1 treatment, exhibiting an average value of 3908.0 ± 44.0.

### 2.4. Biochemical Parameters

**Figure 2:**
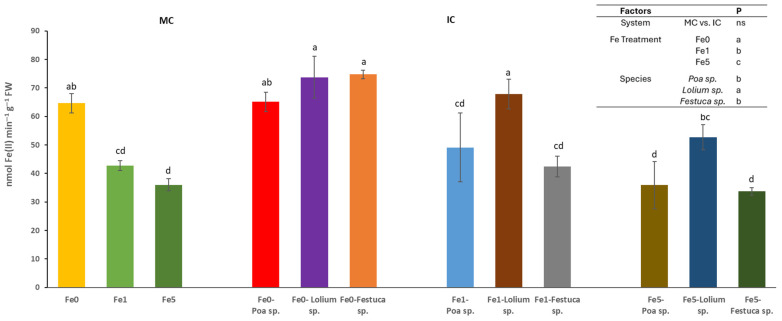
Root ferric chelate reductase activity (nmol Fe(II) min^−1^ g^−1^ FW) determined in at least 6 root tips of *Brassica* sp. considering treatments effects (Fe level: Fe0, Fe1 and Fe5), growing system (MC-Monocrop and IC-Intercrop) and species (*Poa* sp., *Lolium* sp. and *Festuca* sp.), determined at the end of the experiment. For each column, values (mean and standard errors) with different letters were significantly different at *p* < 0.05 (Duncan test).

The cultivation system (MC or IC) had no significant effect on the activity of the ferric chelate reductase (FCR) activity in root tips. However, it was found that differences in Fe levels and species could trigger significant changes (Figure 2). In relation to the effect of Fe availability within the nutrient solution, it was observed that Fe0 treatments yielded significantly higher enzyme activity values in comparison to the Fe5 treatments, with the latter exhibiting significantly lower enzyme activity values. It was observed that *Brassica* sp. plants cultivated in the absence of Fe (Fe0) and IC with *Festuca* sp. or *Lolium* sp. exhibited higher root FCR activity. A similar outcome was observed in plants grown in the Fe1 treatment and IC with the *Lolium* sp.

The catalase activity values in the young leaves of the *Brassica* sp. plants were found to be higher (ranging from 22 to 26 U g^−1^ min^−1^ FW) in the Fe5 treatment group cultivated in both the MC and IC systems, than in the other treatments, which ranged from 3.5 to 10.7 U g^−1^ min^−1^ FW (Table 3). However, when considering the roots, significantly higher values were registered for *Brassica* sp. plants grown without Fe when intercropped with the *Lolium* sp. (25.1 ± 0.4 U g^−1^ min^−1^ FW) or *Festuca* sp. (25.4 ± 0.54 U g^−1^ min^−1^ FW), as well as for the Fe5 treatment of the MC system (24.1 ± 3.2 U g^−1^ min^−1^ FW).

The protein content of the young leaves of the *Brassica* sp. plants was found to be significantly higher in the Fe1 treatment of the plants grown in intercropping with the *Poa* sp. (90.3 ± 0.5 µg BSA). In roots, these values were higher if Fe0 *Brassica* plants were grown intercropped with *Poa* sp. (60.0 ± 0.9 µg BSA) or *Lolium* sp. (61.5 ± 0.9 µg BSA), as well as in plants grown in MC in the Fe5 treatment (61.8 ± 1.1 µg BSA).

*Brassica* plants grown with 5 µM of Fe in the nutrient solution (Fe5), and regardless of the intercropped species, showed higher concentration of phenols in their leaves. However, the plants grown in Fe0 treatments also exhibited significantly higher phenol values when intercropped with the *Poa* sp. (104.4 ± 1.1 mg GAE/L). Interestingly, the concentration of phenols in the roots of *Brassica* sp. were always significantly higher if *Poa* sp. plants were present, particularly under conditions of low Fe levels (Fe0 and Fe1) in the nutrient solution.

### 2.5. Mineral Composition

The concentrations of Ca and S did not differ significantly between treatments, and the interaction of species and system was not significant (Table 4). The K values in *Brassica* sp. plants were found to be significantly higher when they were growing in Fe0 with *Festuca* sp. (60 g kg^−1^). In Fe0 treatment, *Brassica plants* exhibited higher values of Mg when grown with *Lolium* sp. or *Festuca* sp. plants respectively, 10.0 and 9.1 g Mg kg^−1^. The highest concentrations of P (between 9.4 and 10.5 g P kg^−1^) were recorded when *Brassica* plants were cultivated in total absence of Fe (MC and IC), as well as when intercropped with *Poa* sp. plants in the Fe1 nutrient solution.

As a general trend, *Brassica* sp. plants that grew with no Fe or a lower concentration of Fe (Fe1) showed higher B concentrations, in both MC and IC systems and independently of the species of gramineous considered. A similar trend was observed in the values of Mo and Cu concentrations. The Zn concentration was found to be higher in MC without Fe (106 mg kg^−1^). Interestingly, in all *Brassica* sp. plants intercropped with *Poa* sp. and for all Fe concentrations, Zn concentration was significantly higher, compared to treatments where *Festuca* sp. or *Lolium* sp. were used. Mn concentration was also higher in plants growing with Fe1 and intercropped with *Poa* sp. (149 mg kg^−1^), but statistically similar to the plants cultivated in the MC system (Fe0 and Fe5) or intercropped with *Lolium* sp. under low Fe (Fe1).

PC2 reflected the coordinated increases in concentrations of Mn and Zn in the leaves. This observation can be attributed to the coordinated Fe1-IC treatment of *Poa* plants, with the opposite behavior observed in *Lolium* or *Festuca* plants. Pearson’s correlations between PCA variables are presented in Table S2 and corroborate several significative correlations between nutrients and leaf chlorosis parameters.

**Figure 3:**
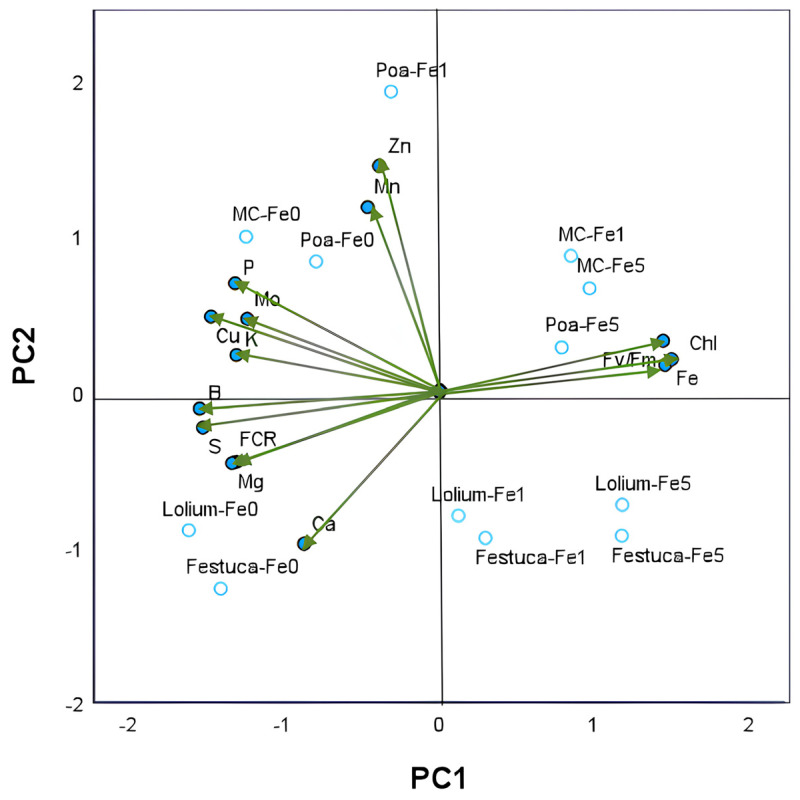
Principal component analysis of nutrient concentrations (in g kg^−1^ DW: P, K, Ca, S and Mg; in mg kg^−1^ DW: Fe, Cu, Zn, Mn, B and Mn) in young leaves of *Brassica* sp. plants (green vectors). PC1—first principal component; PC2—second principal component. Each vector represents the loadings of variables (nutrients and Fe chlorosis parameters: leaf Chl concentration, leaf F_v_/F_m_ and root FCR activity) in each principal component. Loadings represent the relative contribution of each nutrient to that component. Projection of 12 scores resulting from 2 growing systems (monocrop and intercrop) × 3 gramineous species (*Poa* sp., *Lolium* sp. and *Festuca* sp.) × 3 Fe levels (0, 1 and 5 µM of Fe) onto the plane defined by the principal components are also shown. The labels of treatments results from the combination of the letter associated with each gramineous species and the concentration of Fe in nutrient solutions.

Two principal components with eigenvalues greater than 1 were identified amongst the 14 parameters tested using principal component analysis (PCA). These included young leaves nutrient composition, leaf Chl and F_v_/F_m_, and root FCR activity. The cumulative percentage of variance of the two principal axes was found to be 82.5% of the total variance (Table S1). The variations in nutrient concentrations observed in leaves across the different treatment groups yielded a dominant first principal component (PC1), which accounted for 65.1% of the total variance (Figure 3).

The second component, PC2, was found to account for 17.4% of the variance, with subsequent components explaining less than 8%. The results of the analysis indicated a two-dimensional representation of the data, whereby increases in leaf Fe concentration along PC1 were found to be coordinated with leaf Chl and F_v_/F_m_ and placed in contrast to root FCR activity and the nutrient concentration in leaves of K, Mg, P, Cu, S and B. PC2 reflected the coordinated increases in concentrations of Mn and Zn in the leaves, this observation can be attributed to the coordinated Fe1-IC treatment of *Poa* plants, with the opposite behavior observed in *Lolium* or *Festuca* plants. Pearson’s correlations between PCA variables are presented in Table S2 and corroborate several significative correlations between nutrients and leaf chlorosis parameters.

## 3. Discussion

Intercropping is most advantageous when each species can maximize cooperation and minimize competition in both space and time [22]. In general, intercropping with grass plants is a practice that can result in a more sustainable and productive agricultural system in the long term. There are many studies that have reported the benefits of intercropping vegetables with grass species [19,21,24,28,29]. In the present study, *Brassica* sp. plants were cultivated in an intercropping system with three perennial grasses (*Poa pratensis* L., *Lolium perenne* L. and *Festuca rubra* L.) under different Fe levels.

Given the low mobility of Fe in the plant, the first symptoms of iron chlorosis appeared in *Brassica* sp. that grew in the absence of Fe and were characterized by the appearance of a fine reticulate pattern on the new leaves, in which the limbus turned yellow, and the veins remained green [2]. It was observed that the absence of Fe (in MC system) affected the accumulation of biomass in chlorotic plants, since the DW of the aerial part, as well as the height of the plant and the number of leaves were significantly lower compared to plants grown in a MC system with 1 µM and 5 µM of Fe in the nutrient solution.

In an IC system, greater root development of the main crops intercropped has been observed [30,31]. In a study by Wang et al. [31], intercropping maize and wheat was observed to significantly promote the root growth in maize, as evidenced by an increase in the root surface density, compared to plants grown in a MC system. In the present experiment, *Brassica* sp. plants intercropped with *Lolium* sp. or *Festuca* sp. also exhibited higher root DW than plants grown in MC system, in both Fe treatments (Fe1 and Fe5). However, the effect of intercropping on the root to shoot ratio was not entirely consistent with the observations by Zhu et al. [22] in *Brassica* sp. intercropped with barley. Nutritional deficiencies, such as N, P or K, normally reduce root development, but these changes are variable and depend on the nutrient and the parameter under study [32]. In Fe-deficient conditions, root laterization and swelling may occur in tomato plants [33]. However, in strawberry plants, although shoot growth was affected, the root-to-shoot ratio remained unaffected [34].

It is widely reported that low Fe availability in the plant leads to a decrease in total leaf Chl concentration over the test time, thus confirming the importance of Fe in Chl biosynthesis [1,35]. The pronounced decline in Chl concentration observed in young leaves deprived of Fe (Fe0) and the subsequent manifestation of Fe chlorosis substantiate the limited resilience of *Brassica* sp. to Fe deficiency. One of the most significant outcomes of the present study was the potential for *Poa* sp. to mitigate the decline in Chl levels in the leaves of *Brassica* sp. At the end of the experiment, the *Brassica* sp. plants grown without Fe and in association with *Poa* sp. exhibited 38% of their initial Chl, while the Chl of the other treatments demonstrated a more pronounced decrease. A similar trend was observed in Fe1, with *Brassica* sp. plants grown in association with *Poa* sp. (IC-Fe1-*Poa*) exhibiting 20% greater total Chl content than plants grown in monoculture (MC-Fe1) after 13 days of experiment. The effect of intercropping with *Poa* sp. may be related to the complexation of other nutrients that promote the dissipation of stress-induced oxidant power [19] found an increase in Chl values in ‘Swingle’ citrumelo plants when intercropped with *Festuca* sp. and *Poa* sp. in a nutrient solution, and this effect may be attributed to the ability of the compounds released by the grasses to mobilize iron, while [36] also found similar results when intercropping vines with *Festuca* sp. but under field conditions. In fact, considering the restricted mobility of Fe in plants [1] and the total dependence of young leaves on Fe supplied by the roots, the lack of a significant increase in total Fe concentrations under IC conditions was expected due to the absence of this element in the nutrient solution.

The F_v_/F_m_ parameter is indicative of the percentage of energy used in the photochemical pathway, thus providing a definition of the efficiency of photosystem II [37]. In this experiment, it was observed that *Brassica* sp. plants grown without Fe in the nutrient solution had a lower F_v_/F_m_ value. These results were in agreement with those of other studies [37,38,39] which state that in more severe chlorotic states, given the decrease in photosynthetic pigments (chlorophylls a and b), there is also a decrease in PSII efficiency. It is interesting to note that in the present experiment, the F_v_/F_m_ values of *Brassica* sp. plants grown on low Fe level (Fe1) but intercropped with *Poa* sp. were similar to those of the Fe5 treatments, irrespective of the grass species intercropped.

*Brassica* plants grown in the absence of Fe (regardless of the system) showed higher root activity of the FCR enzyme in comparison to those cultivated with other levels of Fe (Fe1 and Fe5). Under conditions of Fe deficiency, there is a process of induction of the expression of the FRO gene, which encodes the FCR enzyme. This results in increased production of the enzyme, which through the process of NADPH-dependent reduction transforms Fe^3+^ into Fe^2+^ [34]. This response, recognized as Strategy I in dicotyledonous plants [40], serves to enhance the availability of metabolically active Fe(II) in the roots. Furthermore, morphological adaptations were observed at the root level, including an increase in the number of lateral apices with a dilated subapical zone. This morphological change may enhance the number of reduction points in the roots, thereby facilitating greater Fe absorption [26,33]. The elevated root FCR activity exhibited by *Brassica* sp. cultivated intercropped with *Festuca* sp. or *Lolium* sp. in the absence of Fe was found to be associated with an increase in lateralization and secondary root formation, as reported by Van Doorn [41] in tomato plants. This result was also recorded in *Brassica* plants grown in with *Lolium* sp., but with only 1 µM of Fe in the nutrient solution. This finding indicates that any compounds released by the grass did not interfere with the response normally seen in Strategy I plants, which is consistent with an increase in root FCR activity under conditions of Fe deficiency. Conversely, the Fe stress signal was not evident in *Brassica* sp. intercropped with *Poa* sp. or *Festuca* sp. grown at low iron concentrations (Fe1).

The first line of defense against the increase in ROS is the presence of enzymes that degrade reactive oxygen species (ROS), such as superoxide dismutase (SOD), which converts superoxide into hydrogen peroxide; catalase (CAT), ascorbate peroxidase (APX) and glutathione peroxidase (GPX), which convert hydrogen peroxide into water [41]. The second line of defense is the presence of endogenous antioxidant substances, such as phenols and anthocyanins and others like flavonoids and carotenoids [41,42]. Catalase is an important enzyme in the antioxidant system of plants and is mainly located in peroxisomes, organelles that multiply during exposure to stress [43,44]. Consequently, when plants are subjected to low levels of Fe, catalase activity increases in response to oxidative stress, depending on the organ in question. Cui et al. [45] recorded a 30% increase in catalase activity in the roots of soya plants grown in the absence of Fe compared to plants grown with Fe in the nutrient solution. In a study conducted by Gama et al. [34], it was observed that the activity of the enzyme catalase did not undergo a notable change in new citrus leaves exhibiting normal, mild, and moderate chlorosis. However, a significant increase was noted in new leaves displaying severe chlorosis. Alfalfa plants grown in consociation with *Lolium* sp. in environments contaminated with heavy metals also showed higher values of catalase activity in both the root and aerial parts [45].

In our experiment, the response to oxidative stress via catalase varied depending on the type of organ considered. The analysis of the dynamics of the enzyme in the roots of plants grown without iron (Fe0) suggests that the interaction with *Poa* sp. may have inhibited the oxidative stress signal; however, the observed value in Fe0 plants in MC remains to be explained. Curiously, this pattern was not observed in the young leaves of *Brassica* sp. where the highest values were recorded in the plants grown in Fe5.

The antioxidant effect of polyphenols is associated with their direct free radical scavenging activity, which is a consequence of their capacity to chelate oxidized transition metal ions [41,46]. The results of the trial seem to demonstrate that IC with *Poa* sp. promoted the production of phenols in the roots of *Brassica* sp. plants, irrespective of the Fe availability in the solution. A similar trend was observed in the young leaves, albeit exclusively in the absence of iron. This outcome aligns with the findings reported by Bostan et al. [47], who observed that an extract of *Poa pratensis* L. was characterized by a high concentration of allelopathic compounds, including phenolic compounds. The good performance of *Brassica* sp. plants intercropped with *Poa* sp. are consistent with the findings reported by Saavedra et al. [27]. In this study, root exudates were analyzed in *Poa* sp., *Festuca* sp. and *Lolium* sp., and several compounds were identified, namely carboxylic acids, phenolic acids, polyphenols and peptides. In particular, *Poa* plants cultivated in the absence of Fe or under conditions of low Fe concentration have been observed to secrete 3-hydroxy-3-methylglutaric acid. Glutaric acid and its derivatives have been demonstrated to play a regulatory role in the activity of ROS, a factor which may elucidate the observed results in relation to catalase activity. The decrease in Fe values observed in new leaves of *Brassica* sp. grown in the absence of Fe is consistent with the findings reported in the literature for citrus [48], strawberry [26,34] and *Prunus* [49] plants.

The absence of Fe in the nutrient solution did not affect K levels in new leaves but led to increased concentrations of P, Zn, and Cu. This may result from the substitution of Cu for Fe due to their physiological interaction [50,51,52,53]. Additionally, the increase could be attributed to a “concentration effect” caused by reduced biomass in Fe-deficient plants.

Intercropping has been demonstrated to enhance the uptake of P. The inter-specific uptake of P was prominent when grasses were intercropped with vegetables, especially fava bean [54] and promoted the uptake of K [55].

In this experiment, it was observed that *Brassica* sp. plants associated with *Poa* sp. accumulated more Zn compared to those associated with *Festuca* sp. or *Lolium* sp. This result aligns with the findings of [56], who reported that Fe deficiency leads to excessive Zn absorption, resulting in nutrient stress that inhibits plant growth, as observed in *Zea mays*. Similarly, Ref. [14] demonstrated that Fe deficiency in *Nicotiana tabacum* L. significantly increased the accumulation of competitive metals such as Zn, Cu and Mn.

Several studies show that compounds released by grasses have an affinity for binding to micronutrients other than Fe, such as Zn and Mn [57,58,59]. As with Fe, Zn is a transition metal that participates in a multitude of metabolic processes, including amino acid synthesis and photosynthesis. The relationship between the accumulation of Zn in the young leaves of *Brassica* sp. in the presence of *Poa* sp. (and to a lesser extent, also for Mn) seems to be one of the most important results.

In addition to being an enzyme activator, Mn is also an important constituent of photosystem II [1], so it is likely that the higher total Chl value observed at the end of the experiment in plants grown in association with *Poa* sp. compared to those grown in the MC system and associated with the other grasses. This is probably due to the greater uptake of Zn and Mn associated with the enhanced uptake of polar metabolites, such as sugars, organic acids, lipids and amino acids [20].

In conditions of Fe deficiency (Fe0) or a low level of Fe (Fe1), *Brassica* sp. has been observed to accumulate Cu in the new leaves. The Cu concentrations observed were consistently higher irrespective of the cultivation system or grass species used. This phenomenon may be due to the physiological interaction between Fe and Cu, where Cu has been observed to be a substitute for Fe in its absence [50,51,52,53]. Additionally, the possible “concentration effect” resulting from reduced plant biomass under Fe deficiency cannot be excluded. At the conclusion of the experiment, an increase in the uptake of metals (Cu, Zn, and Mn) was observed in place of Fe, accompanied by an enhancement in P accumulation in plants grown without Fe. This observation may be attributed to the accumulation of P compounds, such as phytic acid, which plants use for the purpose of storing P. PCA analysis revealed distinct behaviors among plants subjected to varying levels of Fe, indicating clear separations in plant response due to Fe deficiency.

From a global perspective, it is possible to indicate that the association between *Brassica* sp. and *Poa* sp. at limiting iron levels (Fe1) stands out as the most effective combination, promoting Fe utilization. This is evidenced by higher chlorophyll and photosynthetic efficiency values, as well as an increase in the leaf levels of Zn and Mn.

## 4. Materials and Methods

### 4.1. Plant Growth Conditions

An experiment was conducted in nutrient solutions, using an intercropping system comprising *Brassica* sp. *oleracea* cv. ‘Italica’ and three different gramineous species: *Festuca rubra* L. (red fescue), *Poa pratensis* L. (blue grass) and *Lolium perenne* L. (perennial ryegrass). The plants of *Brassica* sp. and the seeds of the gramineous species were purchased from a commercial nursery.

Seeds were germinated and grown for two weeks on vermiculite in a greenhouse under conditions of natural photoperiod conditions (October–January), with an air temperature ≤25 °C and an average relative humidity of 65%. During this period, the seedlings were watered and irrigated once with a half strength Hoagland nutrient solution, which provided an adequate supply of Fe (5 µM of Fe applied as Fe(III)-EDDHA).

**Figure 4:**
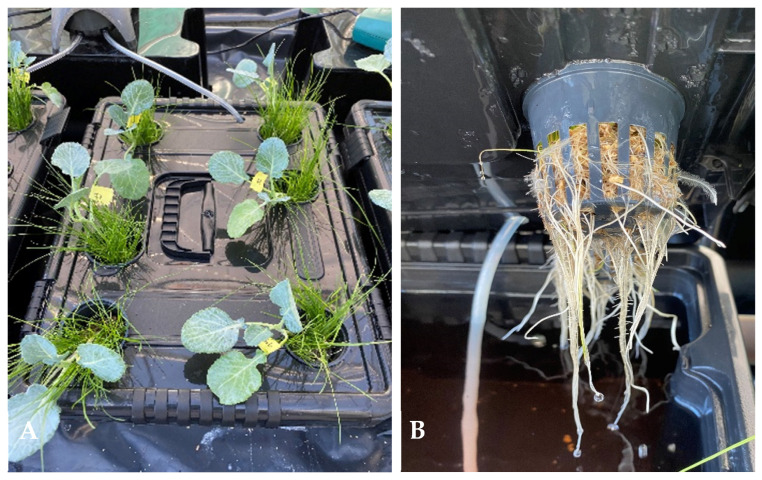
Illustration of the intercropping system utilized in the experimental setup. The cultivation of *Brassica* sp. and *Festuca* plants (**A**) is shown, along with a detailed representation of the intricacies of the root system (**B**). These plants were cultivated in a nutrient solution with 5 µM of Fe within a 12-L vessel.

At the time of transplantation, the *Brassica* plants exhibited analogous physiological conditions (with a mean height of approximately 10 cm and leaf chlorophyll of 861 ± 24 µmol m^−2^). Before the *Brassica* sp. plants were placed in each treatment, the roots were disinfected by immersion in a solution containing 2 g L^−1^ of fosetyl aluminum (C_6_H_18_AlO_9_P_3_) for a period of two hours, followed by washing with tap water. The *Brassica* sp. plants were then cultivated either as monocrop (MC) or intercrop (IC) conditions, under three different levels of Fe in the nutrient solution: with no Fe supplementation (Fe0), with 1 µM of Fe (Fe1) acting as a low concentration of Fe, and with 5 µM of Fe (Fe5) acting as a positive control. The levels of Fe (Fe1 and Fe5) were added as Fe(III)-EDDHA, a commercial fertilizer with 6% of Fe whose effectiveness has been well-documented. Following the filling of each box with 12 L of Hoagland’s nutrient solution, the treatments were established by pipetting the following amounts of Fe (mL) from a 100 mM stock solution of Fe. The quantities of Fe0, Fe1 and Fe5 contained within the box are 0 mL, 1.2 mL and 6 mL, respectively. The total number of treatments was 12, comprising MC-Fe0, MC-Fe1, MC-Fe5 for *Brassica* alone (three treatments), and IC-Fe0, IC-Fe1 and IC-Fe5 for each of the three grass species (*Poa* sp., *Lolium* sp. and *Festuca* sp.—three treatments for each species). In the IC system, each *Brassica* plant was cultivated alongside a group of grass plants weighing 30 g of fresh weight (FW). A minimum of six *Brassica* plants were utilized for each treatment. The experiment was conducted in 12-L polythene containers filled with a complete Hoagland solution (Figure 4A,B), which contained the following concentrations (in mM): 5.0 Ca (NO_3_)_2_.4H_2_O, 5.0 KNO_3_, 1.0 KH_2_PO_4_, 2.0 MgSO_4_. and (in μM): 46.0 H_3_BO_3_, 0.8 ZnSO_4_.7H_2_O, 0.4 CuSO_4_.5H_2_O, 0.9 MnCl_2_.4H_2_O and 0.02 (NH_4_)_6_Mo_7_O_27_. The concentrations indicated were obtained by dissolving stock solutions made on a 1 M basis.

The initial pH of the nutrient solutions was adjusted to 6.0 ± 0.2 using NaOH 0.1 M and the electrical conductivity (EC) was 2.1 ± 0.1 dS m^−1^. No further pH adjustments were made. The pH and EC of the solutions were monitored at two-day intervals, but no significant changes were observed (less than 5%). The nutrient solution was subjected to frequent aeration, by alternating cycles of 15 min with and without aeration, programmed with a timer.

The methodology used for the experiment setup was already described by Saavedra et al. [28].

### 4.2. Leaf Chlorophyll (Chl) Concentration

Leaf chlorophyll (Chl) concentrations are directly related to the degree of iron chlorosis and were estimated three times per week, using a portable SPAD-502 apparatus (Minolta Corp., Osaka, Japan). In order to convert the SPAD-502 values into Chl per unit of leaf area (μmol m^−2^) of *Brassica* sp. plants, a calibration curve was obtained in a preliminary experiment using plants exhibiting varying degrees of chlorosis. In brief, foliar discs exhibiting varying different degrees of Fe deficiency as determined by the SPAD-502 were extracted from the same leaf area with 100% acetone in the presence of Na ascorbate [2]. Subsequently, the extracted pigments were quantified spectrophotometrically according to the methodology proposed by Lichtenthaler [60]. The relationship between Chl and SPAD-502 readings (SPAD) was found to be:

Chl = 0.083 × (SPAD) 2 + 10.04 × SPAD + 67.44

with an R^2^ = 0.95 (*p* < 0.001) and a sample size of 28.

### 4.3. Root Ferric Chelate Reductase (FCR) Activity

The efficiency of plants in acquiring Fe can be understood by measuring the activity of the FCR. Bathophenanthroline disulfonate (BPDS), which forms a red Fe_3_BPDS_2_ complex with Fe(II), was used to measure the activity of the FCR (EC.1.16.1.17) in the root tips of *Brassica* sp. plants at the end of the experiment, as previously described by Bienfait et al. [61]. In summary, a single root tip measuring approximately 2 cm was gently washed with distilled water and excised with a razor blade from at least three *Brassica* sp. plants of each treatment. Each root tip was incubated in an Eppendorf tube for 1 h in the dark with 900 μL of micronutrient-free Hoagland’s half-strength nutrient solution containing 300 μM BPDS, 500 μM Fe(III)-EDTA and 5 mM of MES buffer pH 6.0. A spectrophotometer (CADAS 100 UV-VIS Photometer; Dr. Lange, Düsseldorf, Germany) was then used to quantify FCR activity at 535 nm. An extinction coefficient of 22.14 mM^−1^ cm^−1^ was used to correct for non-specific Fe reduction, and blank controls without root segments were also used. Roots were gently dried with blotting paper and FW was determined. Blank controls without root tips were also used to correct for any non-specific photoreduction. Root FCR activity was expressed on the basis of FW.

### 4.4. Chlorophyll Fluorescence

A quantitative measure of the photochemical and non-photochemical energy dissipation processes occurring in chlorotic leaves can be obtained using the non-invasive and non-destructive technique of Chl *a* fluorescence [62]. Chl *a* fluorescence parameters were measured with a non-modulated fluorimeter (PEA, Plant Efficiency Analyzer, Hansatech, Kings Lynn, UK) at the conclusion of the experiment in the second fully developed leaves of *Brassica* sp. plants of each treatment. After a period of at least one hour of pre-adaptation to darkness, a saturation pulse of five seconds and a photosynthetic photon flux density (PPFD) of 3000 µmol m^−2^ s^−1^ were applied, after which the following parameters were determined: basal fluorescence (F_0_), maximum fluorescence (F_m_) and variable fluorescence (F_v_ = F_m_ − F_0_). The maximum photochemical efficiency of photosystem II was estimated through the variable-to-maximum Chl *a* fluorescence ratio (F_v_/F_m_). All measurements were performed at room temperature.

### 4.5. Biomass and Leaf Mineral Composition

At the end of the experimental period (day 36), a minimum of six *Brassica* sp. plants per treatment were harvested. Shoots (stem and leaves) and roots were considered for biomass parameters. Plant mineral composition was determined in young leaves. Initially, the young leaves were first washed with tap water and then with a deionized water solution containing a non-ionic detergent at a concentration of 0.1% to remove any surface contamination. Following this, the young leaves were washed with a 0.01 M HCl solution. After this, three rinses were conducted with distilled water, and the fresh weight (FW) was determined. The dry weight (DW) was estimated after drying at 60 °C until a constant weight was achieved. The dried samples were weighed and then digested in an acidic solution containing 1 M HCl and 1 M HNO_3_. The concentration of K, Ca, Mg, P, S, Fe, Cu, Zn, Mn, B, and Mo was determined by inductively coupled plasma with optical emission spectroscopy detector (ICP-OES, 7000 series, Agilent Technology, Santa Clara, CA, USA) following standard laboratory procedures [63].

### 4.6. Catalase Activity, Total Protein and Total Phenolic Contents

Iron deficiency can cause oxidative stress, prompting plants to activate protective mechanisms that scavenge reactive oxygen species. To determine the catalase activity (EC 1.11.1.6) as well as the total protein, and total phenolic contents, a single extraction was performed [64]. In summary, a total of three *Brassica* sp. plants were randomly selected from each treatment at the conclusion of the experiment. The plants were then washed and frozen at a temperature of −80 °C until the analysis was performed as described below. Approximately 0.50 g of young leaves and 0.50 g of root tips were weighed and macerated with 2 mL of the extraction solution and 0.02 g of insoluble polyvinylpolypyrrolidone (PVPP) (1% *w*/*v*) in an ice-frozen mortar until a homogeneous extract was obtained. The plant extracts were centrifuged at 12.000 rpm for 20 min at 4 °C, after which the supernatant was removed for the remaining biochemical determinations. The extraction solution consisted of Tris-HCl (100 mM C_4_H_11_NO_3_, pH 7.5), DTT (1,4-Dithiothreitol; 1.5 mM C_4_H_10_O_2_S_2_), EDTA (1 mM C_10_H_16_N_2_O_8_) and Triton 100 (0.1% *w*/*v*).

The activity of catalase was quantified by measuring the decrease in the concentration of hydrogen peroxide (H_2_O_2_) in the solution [65]. Briefly, 50 µL of a 130 mM H_2_O_2_ standard solution was mixed with 50 µL of each sample (supernatant) for 5 min at 25 °C, after which sodium azide (15 mM NaN_3_) was added, and the catalase activity was measured colorimetrically at 520 nm by adding DHBS (3,5-dichloro-2-hydroxybenzenesulphonic acid), which served as the indicator. In parallel, blank samples were prepared.

The quantification of total proteins was conducted in the same plant extracts, employing the procedure described by Bradford [66]. This method utilizes the Coomassie Brilliant Blue G-250 dye (C.I. 42655) for the binding proteins, which caused a color change from red to blue. The protein concentration was then measured by spectrophotometry at 595 nm, following the establishment of a standard curve with BSA (bovine serum albumin), with blanks used as controls.

The quantification of total phenolic compounds was performed in accordance with the Folin-Ciocalteu method [67]. Each 0.25 mL of plant extract was mixed with 1.25 mL of Folin-Ciocalteu reagent solution (1:10) and incubated for 2 min at room temperature before the addition of sodium carbonate (1 mL of 7.5% *w*/*v* Na_2_CO_3_). Folin-Ciocalteu reagent is a clear yellow liquid that turns blue when it reacts with reducing agents, such as phenols. The resulting mixture was then left to rest for a period of 30 min at room temperature, after which the absorbance at 765 nm was measured. Results were expressed as mg of gallic acid equivalents (mg GAE), using a calibration curve prepared with gallic acid. 

For the colorimetry techniques, a CADAS 100 UV-VIS photometer (Dr. Lange, Düsseldorf, Germany) was used.

### 4.7. Statistical Analysis

The effects of the treatments and main factors (Fe level, growing system and grass species) were evaluated by analysis of variance and the means were compared using the Duncan multiple range test (DMRT) at 5%.

Principal component analysis (PCA) was employed to assess the main nutritional patterns in response to all treatments (2 growing systems × 3 Fe levels × 3 gramineous species). The application of PCA facilitates the discernment of associations within data sets that would otherwise remain concealed when analyzed individually. Each extracted component or factor accounts for a proportion of the total variation observed across all data sets and is associated with an eigenvalue. The eigenvector associated with each eigenvalue is representative of the variance within the variables corresponding to the principal component in question. The eigenvectors can be utilized to calculate new values, termed scores, for each observation on each principal component. The scores can be plotted in order to identify the cases that contributed most to the formation of the component. Only components with eigenvalues greater than one were retained for interpretation of the data (Kaiser’s criterion). To obtain a more accurate representation of gradients in young leaves, nutrients associated with chlorosis variables (leaf Chl concentration and root FCR activity) were obtained through a varimax (normalized) rotation applied to the PCA results.

Statistical analyses were performed using the SPSS^®^ software (IBM SPSS Statistics for Windows, Version 29.0. Armonk, NY, USA, IBM Corp.).

## 5. Conclusions

In summary, in the absence of Fe, plants exhibited lower total Chl values, which are directly related to lower photosynthetic efficiency values, which in turn are related to increased root FCR activities. It can be hypothesized that the different grass species exhibit distinct behavioral patterns when intercropped in combination with *Brassica* plants. For instance, *Brassica* sp. plants intercropped with *Poa* sp. plants demonstrated superior performance in terms of physiological parameters and Fe use efficiency. Conversely, superior vegetative performance was observed when intercropped with *Lolium* sp. or *Festuca* sp. These findings suggest that a complementary intercropping system with *Poa* and one of the other species (*Lolium* or *Festuca*) could be a promising sustainable production system but requires further development.

## Figures and Tables

**Table 1:** The growth parameters (mean ± standard error) of *Brassica* sp. plants in relation to Fe levels: (Fe0, Fe1 and Fe5), the growing system (MC-Monocrop and IC-Intercrop) and the grass species (*Poa* sp., *Lolium* sp. and *Festuca* sp.), determined at the conclusion of the experiment. For each column, means with different letters indicate significant differences between all treatments, as determined by the post-hoc Duncan test (*p* < 0.05). The significance of the interaction between the main factors is also indicated.

Treatment		Plant Height (cm)	Number of Leaves	Dry Weight—DW (g)		Root/Shoot
				Root	Shoot	(DW)
Monocrop—MC						
	Fe0	12.3 ± 0.6 d	7.8 ± 0.3 cd	0.2 ± 0.03 b	1.0 ± 0.1 d	0.3 ± 0.01 bcd
	Fe1	14.2 ± 1.7 c	9.7 ± 0.3 b	0.3 ± 0.03 b	2.0 ± 0.1 c	0.2 ± 0.01 d
	Fe5	15.5 ± 0.5 bc	10.7 ± 0.2 a	0.4 ± 0.02 b	2.4 ± 0.0 bc	0.2 ± 0.02 d
Intercrop—IC						
Fe0	*Poa* sp.	9.8 ± 0.0 e	8.3 ± 0.3 c	0.2 ± 0.06 b	0.9 ± 0.1 d	0.3 ± 0.02 bcd
	*Lolium* sp.	12.3 ± 0.3 d	8.0 ± 0.0 cd	0.3 ± 0.06 b	0.8 ± 0.1 d	0.4 ± 0.10 a
	*Festuca* sp.	12.3 ± 0.4 d	7.3 ± 0.2 d	0.4 ± 0.02 b	1.1 ± 0.1 d	0.3 ± 0.01 abc
Fe1	*Poa* sp.	11.8 ± 0.3 d	7.3 ± 0.3 d	0.3 ± 0.07 b	1.1 ± 0.1 d	0.3 ± 0.04 bcd
	*Lolium* sp.	18.7 ± 0.3 a	9.7 ± 0.3 b	0.8 ± 0.02 a	2.8 ± 0.5 ab	0.3 ± 0.04 bcd
	*Festuca* sp.	18.7 ± 0.4 a	10.7 ± 0.2 a	0.8 ± 0.02 a	2.3 ± 0.3 bc	0.4 ± 0.02 ab
Fe5	*Poa* sp.	16.0 ± 0.3 b	10.7 ± 0.3 a	0.4 ± 0.09 b	1.9 ± 0.3 c	0.2 ± 0.08 cd
	*Lolium* sp.	19.7 ± 0.3 a	11.0 ± 0.0 a	0.6 ± 0.09 a	3.3 ± 0.0 a	0.2 ± 0.03 cd
	*Festuca* sp.	19.7 ± 0.3 a	10.3 ± 0.3 ab	0.6 ± 0.08 a	2.5 ± 0.3 bc	0.3 ± 0.03 bcd
Main factors						
Species × System		**	ns	***	***	*
Species × Fe level		***	***	***	***	***
System × Fe level		***	***	***	***	***
Species × System × Fe level		***	***	***	***	**

ns—not significant: * *p* < 0.05; ** *p* < 0.01; *** *p* < 0.001.

**Table 2:** The mean values (±standard error) of the physiological parameters of *Brassica* sp. plants determined at the end of the experiment, considering the effects of the treatments (Fe level: Fe0, Fe1 and Fe5), the growing system (MC-Monocrop and IC-Intercrop) and the species (*Poa* sp., *Lolium* sp. and *Festuca* sp.). For each column, means with different letters indicate significant differences between all treatments, as determined by the post-hoc Duncan test (*p* < 0.05). The significance of the interaction between the main factors is also indicated.

Young Leaves					
Treatments		Chl	F_0_	F_m_	F_v_/F_m_
Monocrop—MC					
	Fe0	163.1 ± 8.3 g	1475.0 ± 61.0 b	2271.3 ± 110.4 g	0.24 ± 0.02 e
	Fe1	606.2 ± 2.7 d	548.3 ± 8.0 d	3248.0 ± 47.0 de	0.82 ± 0.01 a
	Fe5	761.6 ± 14.0 c	600.9 ± 16.0 d	3455.0 ± 80.0 cd	0.83 ± 0.01 a
Intercrop—IC					
Fe0	*Poa* sp.	368.0 ± 24.0 f	1791.0 ± 165.0 a	2866.0 ± 169.0 f	0.39 ± 0.03 d
	*Lolium* sp.	182.0 ± 16.0 g	465.0 ± 49.1 d	524.0 ± 62.4 i	0.16 ± 0.01 f
	*Festuca* sp.	191.0 ± 23.0 g	424.2 ± 33.1 d	546.0 ± 67.3 i	0.21 ± 0.03 ef
Fe1	*Poa* sp.	577.0 ± 12.0 d	542.0 ± 42.0 d	3581.0 ± 54.0 bc	0.84 ± 0.01 a
	*Lolium* sp.	529.0 ± 21.0 e	1477.0 ± 203.0 b	3908.0 ± 44.0 a	0.59 ± 0.05 c
	*Festuca* sp.	600.0 ± 23.0 d	1490.0 ± 94.0 b	2778.0 ± 31.0 f	0.69 ± 0.04 b
Fe5	*Poa* sp.	919.0 ± 17.0 a	520.0 ± 27.0 d	3053.0 ± 177.0 ef	0.83 ± 0.00 a
	*Lolium* sp.	824.0 ± 4.0 b	1330.0 ± 80.0 b	3807.0 ± 60.0 ab	0.81 ± 0.01 a
	*Festuca* sp.	798.0 ± 10.0 bc	821.0 ± 28.0 c	3843.0 ± 126.0 ab	0.87 ± 0.03 a
Main factors					
Species × System		ns	ns	ns	ns
Species × Fe level		***	***	***	***
System × Fe level		***	***	***	***
Species × System × Fe level		***	***	***	***

ns—not significant: *** *p* < 0.001.

**Table 3:** Enzymatic activities (mean ± standard error; n = 4) of *Brassica* sp. plants considering treatments effects (Fe level: Fe0, Fe1 and Fe5), growing system (MC-Monocrop and IC-Intercrop) and species (*Poa* sp., *Lolium* sp. and *Festuca* sp.), determined at the end of the experiment. FW—fresh weight. For each column, means with different letters indicate significant differences between all treatments, as determined by the post-hoc Duncan test (*p* < 0.05). The significance of the interaction between the main factors is also indicated.

Treatments		Catalase U g^−1^ min^−1^ FW	Proteins µg BSA	Phenols mg GAE L^−1^
Young Leaves				
Monocrop—MC				
	Fe0	10.4 ± 0.6 bcd	63.4 ± 1.7 f	85.1 ± 1.7 f
	Fe1	8.0 ± 0.7 bcde	85.0 ± 1.1 bc	102.7 ± 0.6 ab
	Fe5	24.1 ± 3.2 a	89.0 ± 0.6 ab	96.9 ± 1.5 cd
Intercrop—IC				
Fe0	*Poa* sp.	6.7 ± 0.3 cde	75.6 ± 0.8 d	104.4 ± 1.1 a
	*Lolium* sp.	10.7 ± 0.6 bc	76.4 ± 1.2 d	93.3 ± 1.8 de
	*Festuca* sp.	12.5 ± 1.2 b	69.7 ± 2.1 e	90.1 ± 2.4 e
Fe1	*Poa* sp.	5.2 ± 0.5 de	90.3 ± 0.5 a	99.3 ± 0.5 bc
	*Lolium* sp.	3.5 ± 0.3 e	88.0 ± 0.6 ab	75.0 ± 2.1 g
	*Festuca* sp.	7.9 ± 0.6 bcde	73.3 ± 1.1 de	85.8 ± 2.3 f
Fe5	*Poa* sp.	26.0 ± 2.4 a	82.5 ± 1.8 c	106.0 ± 0.9 a
	*Lolium* sp.	22.0 ± 3.5 a	77.7 ± 3.4 d	104.3 ± 0.8 a
	*Festuca* sp.	24.0 ± 0.5 a	63.6 ± 1.8 f	107.0 ± 1.8 a
Main factors				
Species × System		ns	**	ns
Species × Fe level		***	***	***
System × Fe level		***	***	***
Species × System × Fe level		***	***	***
Roots				
Monocrop—MC				
	Fe0	20.9 ± 0.8 cd	45.9 ± 1.1 cd	17.5 ± 1.2 d
	Fe1	17.5 ± 0.5 e	53.2 ± 2.3 b	15.1 ± 0.4 de
	Fe5	24.5 ± 0.2 ab	61.8 ± 1.1 a	24.7 ± 0.6 bc
Intercrop—IC				
Fe0	*Poa* sp.	22.5 ± 0.5 bc	60.0 ± 0.9 a	27.8 ± 1.8 a
	*Lolium* sp.	25.1 ± 0.4 a	61.5 ± 0.9 a	17.4 ± 0.5 d
	*Festuca* sp.	25.4 ± 0.5 a	45.0 ± 1.1 d	16.9 ± 0.6 de
Fe1	*Poa* sp.	14.5 ± 0.3 f	50.8 ± 1,1 b	27.6 ± 0.5 a
	*Lolium* sp.	10.2 ± 0.5 g	50.1 ± 0.6 bc	23.5 ± 0.6 c
	*Festuca* sp.	21.1 ± 0.9 cd	44.2 ± 1.1 d	14.6 ± 0.6 e
Fe5	*Poa* sp.	19.0 ± 0.7 de	49.6 ± 0.9 bc	26.5 ± 0.1 ab
	*Lolium* sp.	21.2 ± 0.4 cd	41.7 ± 1.5 de	22.8 ± 0.6 c
	*Festuca* sp.	21.5 ± 2.0 c	38.4 ± 2.6 e	25.1 ± 0.4 bc
Main factors				
Species × System		ns	*	***
Species × Fe level		***	***	***
System × Fe level		***	***	*
Species × System × Fe level		***	***	***

ns—not significant: * *p* < 0.05; ** *p* < 0.01; *** *p* < 0.001.

**Table 4:** Nutrient concentrations (mean ± standard error; n = 4) in young leaves of *Brassica* sp. plants considering treatments effects (Fe level: Fe0, Fe1 and Fe5), growing system (MC-Monoculture and IC-Intercropping culture) and Species (*Poa* sp., *Lolium* sp. and *Festuca* sp.), determined at the end of the experiment. For each nutrient, means with different letters indicate significant differences between all treatments, as determined by the post-hoc Duncan test (*p* < 0.05). The significance of the interaction between the main factors is also indicated.

**Treatments**			**K** **g kg^−1^**	**Ca** **g kg^−1^**	**Mg** **g kg^−1^**	**P** **g kg^−1^**	**S** **g kg^−1^**
		Fe0	40 ± 3.7 b	24 ± 3.7 abc	7.2 ± 1.0 cdef	9.4 ± 0.8 abc	19 ± 2.2 ab
		Fe1	31 ± 2.8 b	20 ± 4.2 bc	4.1 ± 0.4 f	7.0 ± 1.0 bcde	13 ± 1.2 ab
		Fe5	34 ± 1.3 b	33 ± 2.8 ab	5.3 ± 0.5 ef	6.9 ± 0.4 bcde	14 ± 1.2 ab
Fe0		*Poa* sp.	43 ± 2.2 b	21 ± 1.8 c	6.4 ± 0.4 cdef	10.5 ± 0.5 a	18 ± 0.6 ab
		*Lolium* sp.	42 ± 7.7 b	34 ± 3.9 a	10.0 ± 0.9 a	8.3 ± 2.3 abcd	19 ± 3.1 a
		*Festuca* sp.	60 ± 12.1 a	36 ± 4.1 a	9.1 ± 1.2 ab	9.5 ± 2.2 ab	19 ± 3.5 ab
Fe1		*Poa* sp.	46 ± 3.3 b	31 ± 2.1 abc	7.3 ± 0.7 bcd	9.8 ± 0.7 ab	18 ± 1.8 ab
		*Lolium* sp.	37 ± 3.7 b	32 ± 2.3 ab	7.8 ± 0.8 bc	5.8 ± 0.9 cde	17 ± 2.1 ab
		*Festuca* sp.	36 ± 2.4 b	35 ± 1.4 a	7.2 ± 0.2 bcde	7.0 ± 0.8 bcde	17 ± 1.1 ab
Fe5		*Poa* sp.	38 ± 1.6 b	31 ± 2.0 abc	5.7 ± 0.5 cdef	7.2 ± 0.6 bcde	12 ± 1.2 b
		*Lolium* sp.	32 ± 1.3 b	26 ± 3.0 abc	5.3 ± 0.5 def	5.2 ± 0.4 de	13 ± 1.0 ab
		*Festuca* sp.	36 ± 1.8 b	25 ± 2.3 abc	5.6 ± 0.6 cdef	4.7 ± 0.4 e	13 ± 1.5 ab
Species × System			ns	ns	*	*	ns
Species × Fe level			*	**	***	*	*
System × Fe level			**	**	***	**	***
Species × System × Fe level			**	**	***	**	*
**Treatments**		**B** **mg kg^−1^**	**Mo** **mg kg^−1^**	**Fe** **mg kg^−1^**	**Cu** **mg kg^−1^**	**Zn** **mg kg^−1^**	**Mn** **mg kg^−1^**
	Fe0	50 ± 4.3 ab	2.9 ± 0.2 a	37 ± 6.6 ab	15 ± 0.2 abc	106 ± 18.9 a	134 ± 21.0 ab
	Fe1	38 ± 2.5 bc	2.3 ± 0.3 bc	86 ± 33.3 abc	9 ± 1.6 bc	38 ± 4.5 cd	87 ± 14.8 bc
	Fe5	38 ± 1.9 bc	1.7 ± 0.1 d	124 ± 35.3 a	7 ± 0.6 bc	45 ± 4.7 bc	136 ± 16 ab
Fe0	*Poa* sp.	49 ± 2.4 ab	2.2 ± 0.2 bcd	40 ± 5.3 bc	13 ± 0.5 abc	76 ± 5.0 a	90 ± 11.6 bc
	*Lolium* sp.	54 ± 7.0 a	2.3 ± 0.3 abc	39 ± 8.5 c	22 ± 13.3 a	24 ± 8.6 def	124 ± 15.2 ab
	*Festuca* sp.	51 ± 7.7 a	2.4 ± 0.2 ab	31 ± 10.1 c	13 ± 3.5 abc	29 ± 7.2 de	55 ± 10.2 cd
Fe1	*Poa* sp.	45 ± 2.3 abc	2.3 ± 0.2 abc	68 ± 6.0 abc	16 ± 1.6 ab	59 ± 3.6 b	149 ± 21.8 a
	*Lolium* sp.	42 ± 3.5 abc	1.9 ± 0.1 bcd	48 ± 3.7 abc	10 ± 3.5 bc	8 ± 1.8 fg	82 ± 14.2 bcd
	*Festuca* sp.	45 ± 1.8 abc	2.0 ± 0.1 bcd	47 ± 3.6 abc	9 ± 2.5 bc	14 ± 3.1 efg	62 ± 4.0 cd
Fe5	*Poa* sp.	37 ± 1.8 bc	1.6 ± 0.1 d	98 ± 27.2 abc	7 ± 0.2 bc	45 ± 3.0 bc	64 ± 9.1 cd
	*Lolium* sp.	37 ± 3.6 bc	1.6 ± 0.1 d	80 ± 7.8 abc	5 ± 0.8 bc	11 ± 1.2 fg	66 ± 11.6 cd
	*Festuca* sp.	33 ± 3.1 c	1.7 ± 0.1 cd	94 ± 27.5 abc	4 ± 0.3 c	5 ± 2.1 g	35 ± 5.9 d
Species × System		ns	ns	ns	ns	***	***
Species × Fe level		**	***	*	*	***	***
System × Fe level		***	***	**	**	***	***
Species × System × Fe level		**	***	*	*	***	***

ns—not significant: * *p* < 0.05; ** *p* < 0.01; *** *p* < 0.001.

## Data Availability

All data are included in the main text.

## Supplementary Materials

The following supporting information can be downloaded at: https://www.mdpi.com/article/10.3390/plants14142215/s1, Figure S1: Photographs of *Brassica* sp. plants in monoculture (MC) and in intercropping (IC) with *Poa* sp., *Lolium* sp. and *Festuca* sp. in a nutrient solution with different levels of Fe (0, 1 and 5 µM Fe respectively Fe0, Fe1 and Fe5) at the end of the experiment. Figure S2: Photographs of *Brassica* sp. roots in monoculture (MC) and in intercropping (IC) with *Poa* sp., *Lolium* sp. and *Festuca* sp. in a nutrient solution with different levels of Fe (0, 1 and 5 µM Fe respectively Fe0, Fe1 and Fe5) at the end of the experiment. Table S1: Principal component analysis (PCA) loadings of the young leaf nutrient composition and leaf chlorosis parameters for *Brassica* plants grown in association with three grass species in different Fe levels. Chl—Leaf chlorophyll concentration, FCR—ferric chelate reductase and F_v_/F_m_—variable fluorescence and maximal fluorescence ratio in young leaves. Significant values are bold. Table S2: Pearson’s correlations between PCA variables: leaf chlorosis parameters and nutrients in young leaves of *Brassica* plants grown in association with three grass species in different Fe levels. Chl—Leaf chlorophyll concentration, FCR—ferric chelate reductase and F_v_/F_m_—variable fluorescence and maximal fluorescence ratio in young leaves.
