# Supplementary material for: Intercropping with Gramineous Plants in Nutrient Solutions as a Tool to Optimize the Use of Iron in Brassica oleracea
Source: Plants (Basel). 2025 Jul 17;14(14):2215. doi: 10.3390/plants14142215 (PMC12299349; doi:10.3390/plants14142215)
Supplement: Supplementary file 1 [file plants-14-02215-s001.zip › 1. Suplementar-Tables-Intercropping.pdf]

Supplementary Information

Intercropping with gramineous plants in nutrient solution as a tool to optimize the use of iron in *Brassica oleracea*.

T. Saavedra, M. Pestana, J. Costa, P. Gonçalves, D. Fangueiro, J.P. da Silva and P.J. Correia

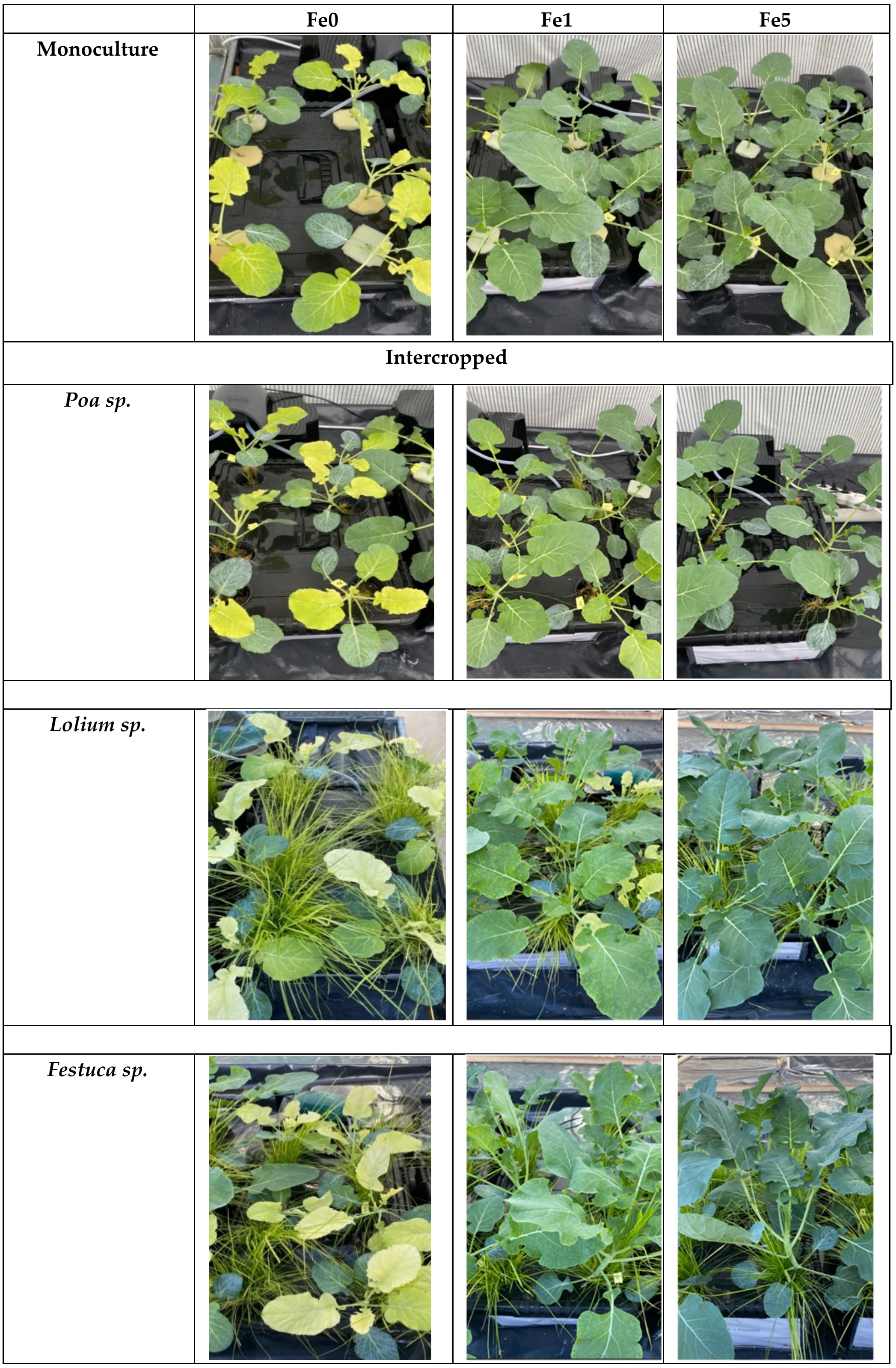

Figure S1: Photographs of *Brassica sp.* plants in monoculture (MC) and in intercropping (IC) with *Poa sp.*, *Lolium sp.* and *Festuca sp.* in a nutrient solution with different levels of Fe (0, 1 and 5 μM Fe respectively Fe0, Fe1 and Fe5) at the end of the experiment.

	Fe0	Fe1	Fe5
**Monoculture**	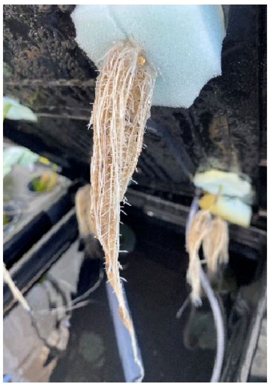	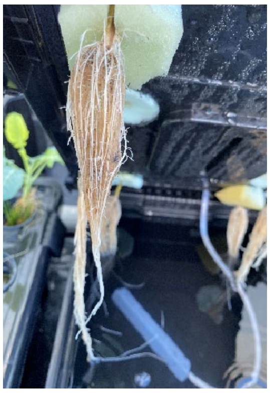	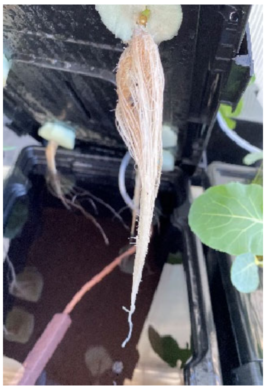
**Intercropped**			
Poa sp.	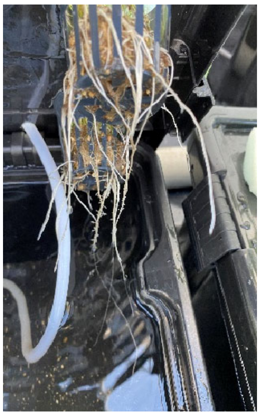	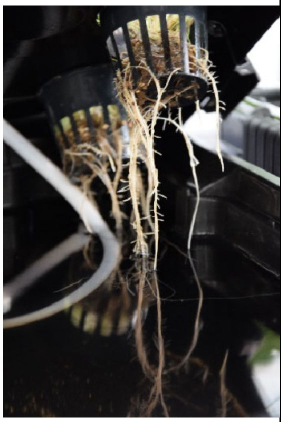	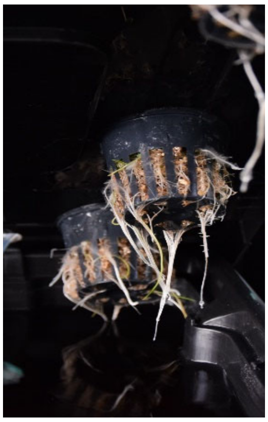
Lolium sp.	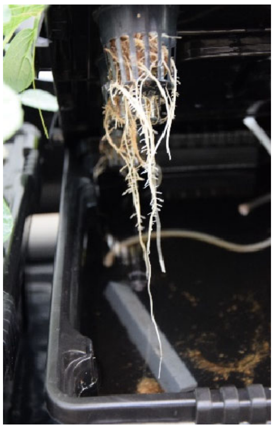	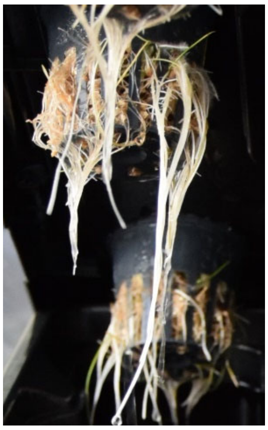	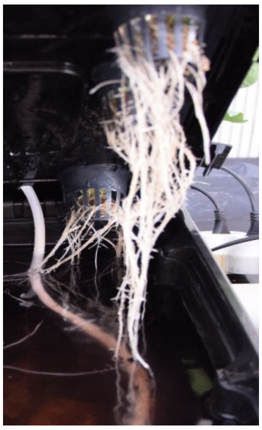
Festuca sp.	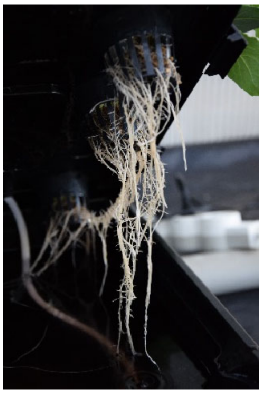	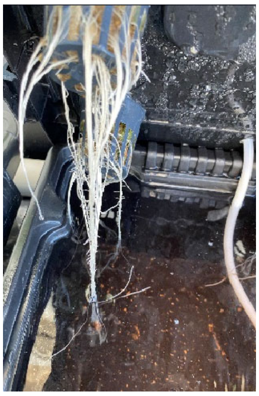	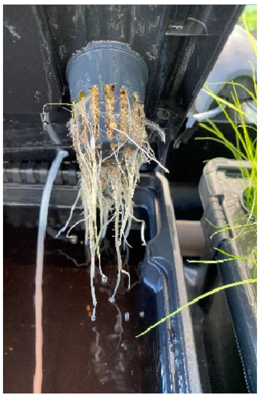

Figure S2: Photographs of *Brassica sp.* roots in monoculture (MC) and in intercropping (IC) with *Poa sp.*, *Lolium sp.* and *Festuca sp.* in a nutrient solution with different levels of Fe (0, 1 and 5 μM Fe respectively Fe0, Fe1 and Fe5) at the end of the experiment.

Table S1: Principal component analysis (PCA) loadings of the for young leaf nutrient composition and leaf chlorosis parameters for *Brassica* plants grown in association with three grass species in different Fe levels. Chl – Leaf chlorophyll concentration, FCR- root ferric chelate reductase and F_v/F_m – variable fluorescence and maximal fluorescence ratio in young leaves. Significant values are bold.

Variables	PC1	PC2
**Chl**	**0.917**	0.106
**F_v/F_m**	**0.909**	0.205
**FCR**	**-0.825**	-0.286
**K**	**-0.825**	0.147
**Ca**	-0.550	-0.619
**Mg**	**-0.842**	-0.292
**P**	**-0.830**	0.439
**Fe**	**0.944**	0.129
**Cu**	**-0.928**	0.303
**Zn**	-0.245	**0.915**
**Mn**	-0.292	**0.747**
**S**	**-0.961**	-0.147
**B**	**-0.974**	-0.070
**Mo**	-0.781	0.294

Table S2: Pearson's correlations between PCA variables: leaf chlorosis parameters and nutrients in young leaves of Brassica plants grown in association with three grass species in different Fe levels. Chl – Leaf chlorophyll concentration, FCR- root ferric chelate reductase and Fv/Fm – variable fluorescence and maximal fluorescence ratio in young leaves.

	**Chl**	**F_v/F_m**	**FCR**	**K**	**Ca**	**Mg**	**P**	**S**	**Fe**	**Cu**	**Zn**	**Mn**	**B**	**Mo**
**Chl**	--													
**F_v/F_m**	0.944**	--												
**FCR**	-0.790**	-0.887**	--											
**K**	-0.649*	-0.616*	0.611*	--										
**Ca**	-0.066	-0.145	0.131	0.407	--									
**Mg**	-0.713**	-0.775**	0.770**	0.711**	0.614*	--								
**P**	-0.692**	-0.605*	0.523*	0.671**	0.007	0.444	--							
**S**	-0.775**	-0.822**	0.804**	0.652*	0.290	0.836**	0.716**	--						
**Fe**	0.765**	0.825**	-0.854**	-0.583*	-0.109	-0.756**	-0.558*	-0.872**	--					
**Cu**	-0.817**	-0.768**	0.730**	0.524*	0.214	0.778**	0.731**	0.847**	-0.699**	--				
**Zn**	-0.441	-0.329	0.170	0.174	-0.385	-0.041	0.741**	0.365	-0.185	0.396	--			
**Mn**	-0.244	-0.190	0.188	0.019	0.059	0.177	0.504*	0.414	-0.021	0.588*	0.613*	--		
**B**	-0.875**	-0.910**	0.848**	0.683**	0.272	0.828**	0.788**	0.938**	-0.859**	0.896**	0.392	0.377	--	
**Mo**	-0.829**	-0.696**	0.591*	0.439	-0.181	0.441	0.709**	0.728**	-0.676**	0.750**	0.620*	0.447	0.728**	--

* Significant difference at $p<0.05$; ** Significant difference at $p<0.01$.
